# Development of a Three-dimensional Printed Emergent Burr Hole and Craniotomy Simulator

**DOI:** 10.7759/cureus.4373

**Published:** 2019-04-03

**Authors:** Nicole Bishop, Darrell Boone, Kerry-Lynn Williams, Roger Avery, Adam Dubrowski

**Affiliations:** 1 Medical Education and Simulation, Memorial University of Newfoundland, St. John's, CAN; 2 General Surgery, Memorial University of Newfoundland, St. John’s, CAN; 3 Neurosurgery, Memorial University of Newfoundland, St. John's, CAN; 4 Emergency Medicine, Memorial University of Newfoundland, St. John's, CAN

**Keywords:** rural healthcare, three-dimensional printing, craniotomy, simulation based medical education

## Abstract

Patients with a traumatic head injury (THI) require immediate surgical intervention, as rapidly expanding intracranial hematomas can be imminently life-threatening, not permitting transfer time to neurosurgical care in a tertiary care center. In rural and remote areas, where neurosurgeons may not be readily available, surgical intervention by Community General Surgeons (CGS) may be required. Currently, the CGS in Newfoundland and Labrador (NL) do not have access to, or have experience training with, an emergent burr hole/craniotomy (EBHC) simulator. One of the barriers is the availability of inexpensive and reliable simulators to practice these skills. Therefore, a low-cost, three-dimensional (3D) printed EBHC simulator was designed and 3D-printed by MUN Med 3D (St John's, NL).

The aim of this technical report is to assess the need for such simulator in rural and remote healthcare centers and report on the iterative development of the EBHC simulator. The 3D-printed EBHC simulator developed by MUN Med 3D was utilized during a general surgery workshop at the 26^th^ Annual Rural and Remote Medicine Conference in St. John’s, NL. A total of six 3D-printed EBHC simulators were provided for the hour and a half workshop. At the end of the workshop, 16 participants were asked to provide feedback on the need for this simulator in their rural or remote environment as well as feedback on the physical attributes. The feedback received from the participants was overall positive, informative, and supported the need for the 3D-printed EBHC simulator.

## Introduction

Patients with a traumatic head injury (THI), such as an expanding epidural hematoma (EDH) or subdural hematoma (SDH), require urgent operative intervention to relieve pressure on the brain and control hemorrhaging [1]. In rural and remote areas, where neurosurgeons may not be readily available, surgical intervention by Community General Surgeons (CGS) may be required [2-4]. For a THI, immediate surgical intervention is essential, as rapidly expanding intracranial hematomas can be imminently life-threatening, not permitting transfer time to neurosurgical care in a tertiary care center [1]. In a case where a patient presents with an EDH or SDH, a CGS is confronted with a difficult decision: operate, in undesirable circumstances in teleconsultation with a neurosurgeon, or transfer the patient to a tertiary care center with the potential for adverse consequences due to delay of care.

While evidence shows that the early decompression and hematoma evacuation of an EDH or SDH improves patient outcomes [1,5-9], Donovan et al. [8] suggest that it is best for this emergency procedure is treated by experienced neurosurgeons in a medical facility equipped with a computed tomography (CT) scanner. In addition, Rinker et al. [1] note that an inexperienced surgeon may encounter difficulties with the emergency burr hole craniotomy and that well-intentioned efforts may actually delay transfer to an experienced neurosurgeon. However, this optimal surgical environment is not readily available in several rural hospitals, as they may not have access to CT scanners and/or have a neurosurgical presence. Therefore, the next best scenario is for a trained-to-proficiency CGS to assess the emergency situation in teleconsultation with a neurosurgeon. If surgical intervention is deemed appropriate due to time-sensitivity, the trained-to-proficiency CGS, remotely assisted by a neurosurgeon, may perform the procedure in a timely matter to prevent progressive neurological impairment or possible death of the patient [1] followed by an emergent transfer to a facility with a neurosurgeon.

Since there are currently a small number of clinical encounters for CGSs to develop skills related to decompression and hematoma evacuation of an EDH or SDH (personal communication with Dr. Darrell Boone and Dr. Michael Parsons), simulation may provide a good training platform to augment the transition from an inexperienced to a trained-to-proficiency CGS. To date, simulation has become an excellent addition to healthcare education, as it promotes skill acquisition and maintenance through hands-on experience [1]. Simulation has been shown to increase the competency and confidence of individuals through the rehearsal of high acuity, low occurrence (HALO) procedural skills, resulting in a reduction of errors [10]. However, currently, the CGSs in rural and remote areas of Newfoundland and Labrador (NL) do not have access to or have experience training with, an emergent burr hole/craniotomy (EBHC) simulator (personal communication with Dr. Darrell Boone and Dr. Michael Parsons). Therefore, the first time they are likely to encounter this situation will be in the emergency setting, highlighting the fact that the CGS need to be skilled but, in most cases, lack the opportunity to develop and maintain these skills.

Subsequently, a three-dimensional (3D)-printed EBHC simulator was designed and printed with the purpose of being incorporated into a simulation-based medical education (SBME) curriculum developed collaboratively by neurosurgeons and CGSs, specifically delivered in rural and remote areas. In this conception, the EBHC simulator is one of the components of a simulation curriculum to train procedural skills related to EBHC. Therefore, the main purpose of this technical report is to assess the need for such simulators in rural and remote areas and report on the iterative development of the EBHC simulator.

## Technical report

Context

In NL, the population (525, 355 as of July 1, 2018) [11] is dispersed over a large geographical area (405,720 square km) [12], with only one tertiary hospital specializing in neurosurgery - the Health Science Centre (St. John’s) [13]. This widely dispersed population leads to long transport times (Table 1) [14-15] that may have potentially detrimental effects on those who have THI, which requires timely, operative intervention.

**Table 1:** Average transfer time from rural communities to a tertiary care center in NL HSC - Health Sciences Centre, the tertiary care center in St. John's, Newfoundland NL: Newfoundland and Labrador

Location	Average Transfer Time to Tertiary Care Center
Happy Valley-Goose Bay (HVGB), Labrador	10 min drive to HVGB airport + 2hr20min flight + 10 min drive to HSC
St. Anthony, Newfoundland	45 min drive to St. Anthony airport + 1hr10min flight+ 10 min drive to HSC
Port aux Basques, Newfoundland	2 hr drive to Stephenville airport + 1hr flight+ 10 min drive to HSC

Inputs

The design time for the simulator (second iteration) was approximately 20 hours with a direct cost of approximately $396.60 (not including overheads). Assembly time for each unit was 40 minutes, with an associated direct cost of $11.90. Note that the design costs are based on a digital modeler time of $19.83/hour and assembly time on a mid-level pay scale for co-op students of $19.83/hour. The total cost of the EBHC simulator, including design time and the materials mention in Table 2 was $440.02. However, this is the cost for the first designed simulator; additional units of the simulator would be costed at materials and assembly only, totaling $44.02.

**Table 2:** Breakdown of the simulator materials, print time, and cost per component

Simulator Component	Material	Layer Height	Infill	Cost
Base	ABS	0.20mm	25%	$5.01
Clamps for base	ABS	0.20mm	25%	$1.23
Brain	TPU (Ninjaflex)	0.15mm	25%	$6.60
Skull	ABS	0.20mm	25%	$4.47
Dural membrane	Latex (latex gloves)	N/A	N/A	$1.50
Blood clots	Silicone Gel	N/A	N/A	$0.25
Skin	Silicone	N/A	N/A	$7.00
Additional hardware to secure the simulator together	1/4"-20 x 1.5" bolts, 1/4" washers, 1/4" nuts, Epoxy	N/A	N/A	$6.06
			Total	$32.12

Process

Simulator

**Figure 1:**
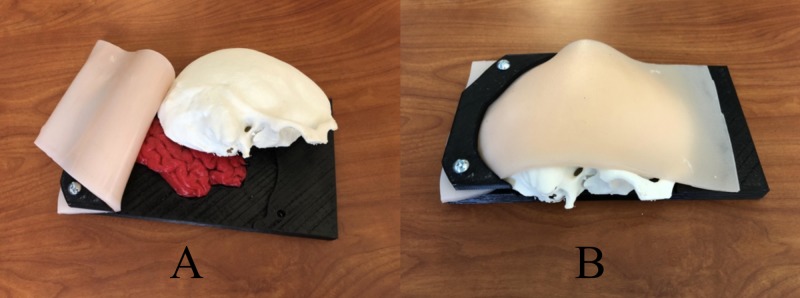
Construction stages of an emergent burr hole/craniotomy simulator A - The first stage of the simulator construction (base, brain, skull, and skin) B - The final stage of the simulation construction (the skin was draped over the skull and secured using the clamps on the base and additional hardware)

The assembly of the 3D-printed EBHC simulators was divided into two categories: SDHs (accumulation of blood between the brain and the dura) [16] and three EDHs (accumulation of blood between the dura and the skull) [17]. Each individual simulator took approximately 40 minutes to construct; from individual printed components to finished product. To construct the EBHC simulator, the brain was inserted into the simulator base (Figure 1a), then depending on epidural or subdural models, the blood clots were placed. For the SDH simulator model, the blood clots were added before the placement of the dura membrane. For the EDH simulator model, the dura membrane was placed on top of the brain, followed by the blood clots. The skull was then placed. The dura membrane and skull were secured to the base using epoxy. Finally, the skin was draped over the skull (Figure 1b) and secured using the clamps on the base and additional hardware.

Need Assessment and Feedback

**Figure 2:**
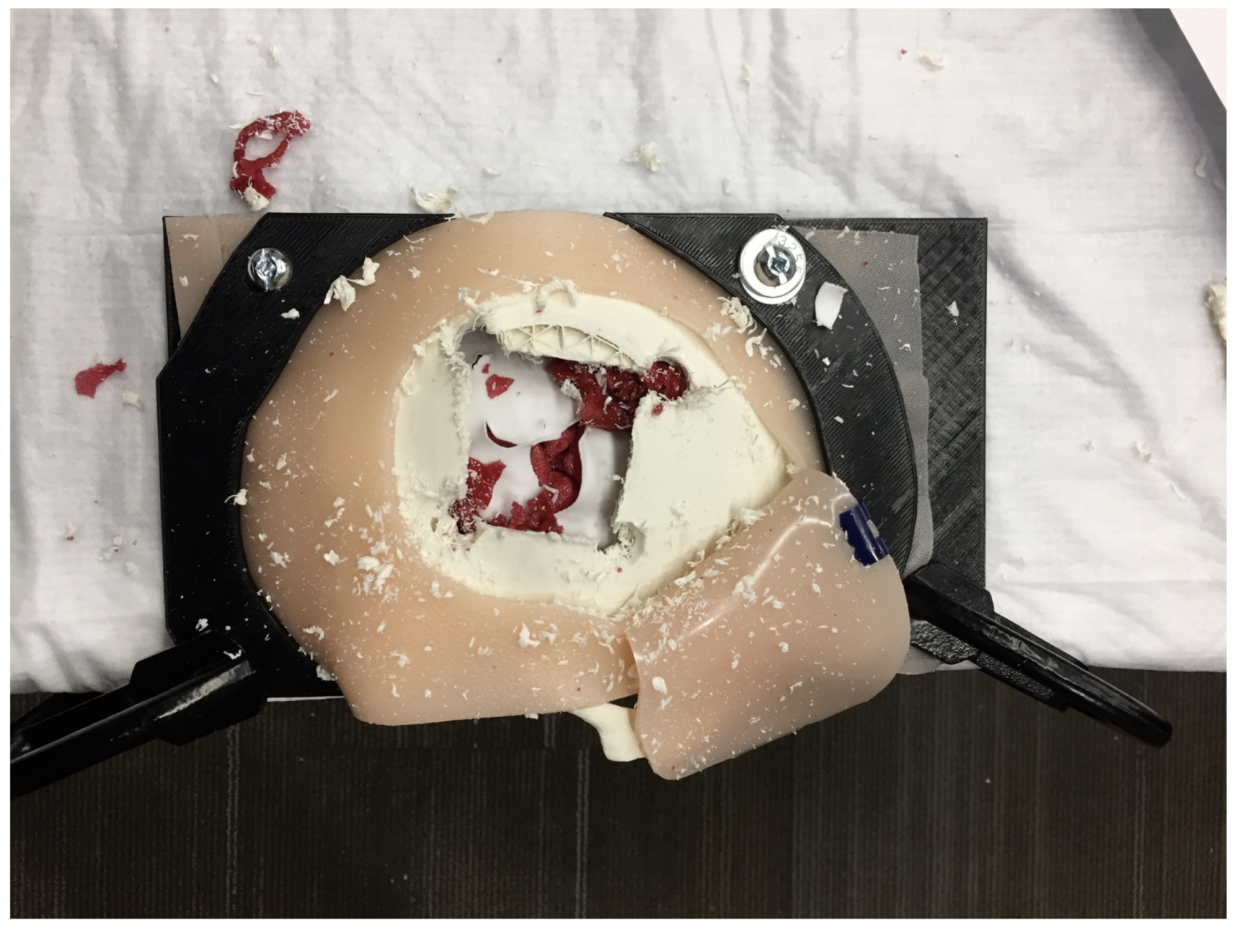
Emergent burr hole/craniotomy simulator after a 15-minute demonstration by an educator

A needs assessment of the simulator for rural and remote training and feedback on the physical attributes of the 3D-printed EBHC simulator were collected after a 1.5 hour, hands-on workshop at the 26th Annual Rural and Remote Medicine Conference in St. John’s, NL. This conference, hosted by the Society of Rural Physicians of Canada, targeted healthcare professionals who are currently practicing, or those who look to practice, in rural and remote areas of Canada. A total of 16 individuals attended the workshop. All individuals indicated that they were rural general practitioners (GP), with two individuals indicating they additionally completed enhanced surgical skills. This sample was used as a convince sample. A local general surgeon (Dr. Darrell Boone) referred to as the educator, led the workshop. The educator began the workshop with a 20-minute PowerPoint presentation describing the emergency burr hole and craniotomy procedures followed by the background on why and how the first iteration of the EBHC simulator was developed (Appendix A). The presentation also informed the participants of the variable transport times from rural areas to tertiary centers within Canada and the implications of these times. After the presentation, an informal 15-minute demonstration on the EBHC simulator was provided by the educator (Figure 2).

Six 3D-printed EBHC simulators were set up on two standard tables, three simulators per table. The participants were given 45 minutes to "play" with the EBHC simulators and were asked to evaluate the realism of the physical attributes of the EBHC simulator (i.e. bone, skin) on the simulator evaluation survey. They were unable to assess the EBHC simulator as an educational tool, as they were not content experts (neurosurgeons and experienced general surgeons) in the specific procedural skills demonstrated.

Outcomes

Nine participants, out of the 15 who attended the workshop completed this survey. The survey posed open-ended questions to receive feedback concerning the need for an EBCH simulator in their practice setting and recommended improvements for future iterations of the physical attributes of the 3D-printed EBHC simulator. It was noted that while THI emergency situations are rare, they can and do occur and multiple participants indicated that they feel simulation training is becoming critical to learning and that they see a need for this skill in their rural and remote environment. Additionally, to the knowledge of the participants, there are no easily reproducible (3D-printed), low-cost simulators they are aware of to practice these procedural skills. Through informal feedback, one participant indicated that the CGS in their rural hospital encountered this emergency situation and that the CGS had no prior practice or experience in the procedures.

Pertaining to the physical attributes of the EBHC simulator, participants indicated that there was a need for blood to be incorporated into the 3D-printed EBCH simulator, and one respondent also provided ideas on how to better secure the skull to the base using gaskets. Furthermore, participants indicated that the texture and feel of the bone were realistic but the skin was too thick to be realistic. The educator also provided additional comments on how to improve the physical attributes of the EBHC simulator. He commented on the fact that the size of the brain was not anatomically correct for the size of the skull that was being used. As well, the colors of the dura membrane and brain were not accurate.

## Discussion

The feedback received from the participants was overall positive, informative, and supported the need for a 3D-printed EBHC simulator in simulation curriculum for rural and remote locations. The implementation of the EBHC simulator into simulation training should focus on systematically addressing who the appropriate learners should be, where the liabilities are, and the relative weight of possible positive outcomes vs complications. With CGSs undergoing appropriate training, the concerns of Rinker et al. (1998) of inexperienced surgeons encountering difficulties with performing emergency burr holes and craniotomies may be addressed [1].

**Figure 3:**
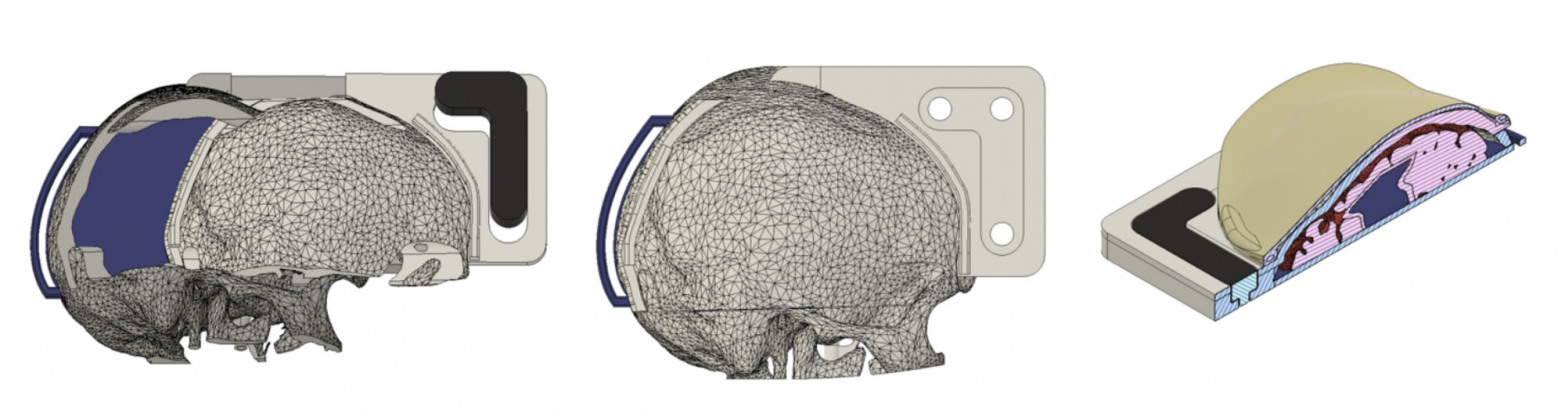
Iterative design after analyzing feedback

The recommendations provided to MUN Med 3D were included in the next iteration of the EBHC simulator (Figure 3). Once the revisions are complete, the task trainer is scheduled to undergo a comprehensive test of face and content validity by experienced content experts (neurosurgeons and experienced general surgeons) to ensure that the task trainer is anatomically correct and provides an overall realistic experience.

## Conclusions

To the author’s knowledge, there are currently no EBHC bench-top simulators on the market and no comparable simulator is incorporated into the current medical curriculum in NL. The current report shows that there is a need for the simulator and that after systematic validation of the 3D-printed EBHC simulator, it has the promise to be a cost-effective addition to an SBME curriculum. Currently, the cost of similar commercially available high-fidelity simulators are magnitudes more expansive, making them potentially prohibitive outside large, well-funded neurosurgical training programs. The developed simulator may fill the identified educational gap and potentially remove the high-risk and high-stress environment of performing these procedural skills for the first time in a real-life situation.
